# Profile of Long-Term Care Recipients Receiving Home and Community-Based Services and the Factors That Influence Utilization in Taiwan

**DOI:** 10.3390/ijerph17082649

**Published:** 2020-04-13

**Authors:** Chia-Mei Shih, Yu-Hua Wang, Li-Fan Liu, Jung-Hua Wu

**Affiliations:** 1 Department of Resources Engineering, National Cheng Kung University, Tainan 701, Taiwan; a0928329323@gmail.com (C.-M.S.); hwaa@mail.ncku.edu.tw (J.-H.W.); 2 Institute of Gerontology, National Cheng Kung University, Tainan 701, Taiwan; m6cj841881@gmail.com

**Keywords:** long-term care, HCBS utilization, aging in place, Andersen health behavioral model

## Abstract

In response to the irreversible aging trend, the Taiwan government has promoted the Long-Term Care (LTC) policy 1.0 launched in 2007 and the LTC policy 2.0 reform since 2016. This study aimed to explore the utilization of formal home and community-based care under LTC policy 1.0 to add scientific support for the on-going LTC policy 2.0 reform. Methods: By using Andersen and Aday’s behavioral model of healthcare utilization, the long-term care dataset was analyzed from 2013 to 2016. A total of 101,457 care recipients were identified after data cleaning. Results: The results revealed that about 40.7% of the care recipients stayed in the care system for more than two years. A common factor influencing the length of home and community-based services (HCBS) utilization period included need factors, where more dependent recipients leave the LTC system regardless of their socio-economic status. However, the utilization period of non-low-income households is significantly affected by the level of service resources. Conclusion: For long-term care needs, the phenomenon of a short utilization period was concerning. This study adds information which suggests policy should reconsider care capacity and quality, especially for moderate to severely dependent recipients. This will allow for better understanding to help maintain care recipients in their own communities to achieve the goal of having an aging in place policy.

## 1. Introduction

Population aging in Taiwan is part of a worldwide phenomenon. According to the World Health Organization (WHO) standards, Taiwan has been an aged society, where the proportion of the population aged 65 and older accounts for over 14% of the overall population since 2018. It may only take eight years for Taiwan to advance from an aged society to a "super-aged society" in 2026, which means the aging rate exceeds 21% [1]. This anticipated period is much shorter than the prediction for other countries like Japan (11 years) and the U.S. (14 years). 

Since life expectancy in Taiwan extended to 80.2 years in 2015 from an estimated healthy life expectancy (HALE) of 71 years in 2014, elderly people may have to rely on others for care for an average of nine years [2]. This situation has caused major fiscal issues and has made sustainability of long-term care a policy priority [3].

### 1.1. Long-Term Care Policy Reform in Taiwan 

Taiwan has a well-known national health insurance system (NHI). In response to the irreversible aging trend, the government has promoted a series of long-term care-related policies, including the LTC policy 1.0 launched in 2007 and the LTC policy 2.0 reform since 2016. The financial subsidies are currently through tax payment, but the country may develop a national LTC insurance system once the service delivery system becomes more complete.

The long-term care plan 1.0 (LTC policy 1.0) promulgated by the Executive Yuan in 2007 was the foundation of a nationwide-subsidized care system for the elderly with limitations on daily living, the disabled over age 50, and aboriginal people over age 55. It covers home nursing, home services, meals and transportation, as well as rehabilitation and respite care services [4]. Among them, home services remained the most commonly used services under the LTC policy 1.0. During the ten-year period in which the LTC policy 1.0 was promoted from 2007 to 2016, resources related to home and community-based services (HCBS) and training programs for caretakers were established. However, the utilization of services was still limited. Statistics showed that the utilization rate of LTC services in the disabled elderly population increased from 2.3% in 2008 to 35.7% in April 2016 [5]. The coverage was apparently insufficient since one of the aims of LTC plan 1.0 was to build an affordable and accessible service system [6]. A reform called the Long-term Care plan 2.0 (LTC policy 2.0) was launched in November 2016, which extended both service items and population coverage and was meant to reinforce the current long-term care system. 

A policy reform should be based on evidence-based research [7]. However, the government did not have enough time to examine the effectiveness of LTC policy 1.0 in all aspects before announcing the LTC 2.0 policy reform. The challenges and problems that the LTC system has faced so far were only mentioned in the approved version of the LTC plan 2.0 (106–115) published by the Ministry of Health and Welfare in 2016. These challenges included limited funding, a shortage of manpower and training, insufficient services and human resources in remote areas, inadequate subsidy quotas and service quality for users, and a lack of caregiver support in the entire system.

Although in the initial stages, policy formulation for health reform was mainly based on colloquial evidence such as experiential knowledge, political decisions, and available resources, evidence-based scientific evidence is critical [7]. A lack of support from an empirical analysis of the HCBS utilization under the era of LTC policy 1.0 could lead to insufficient and inefficient policy reform. Therefore, it is important to examine and understand who uses the HCBS and how they use it. 

### 1.2. Andersen Health Behavioral Model

In order to structurally explore and interpret predictors of service utilization, Andersen and Aday’s behavioral model of healthcare utilization was used for behavior analyses of home and community-based care. The model was initially developed in the late 1960s [8]. It is a multilevel model that incorporates both individual and contextual characteristics related to use of health services, emphasizing the dynamics and feedback among the external environmental, health care systems, personal behavior, and health outcomes [9]. Individual characteristics were divided into three major components: predisposing, enabling, and need factors. 

The term predisposing factors refers to demographic characteristics such as gender, age, and marital status; social factors such as education, occupation, or family status; and mental factors related to beliefs about health. Enabling factors affect an individual’s ability to access available resources including income, health insurance, residential location, and the distribution of medical resources. Need factors include perceived health status, need for health services, and objective measurements of patients’ health status and need for care as performed by professionals [10].

Along with the development of Andersen’s health behavioral model, researchers have applied several factors to expand it, including psychosocial factors [11,12] and health behavior characteristics [13]. Another study added attractiveness variables into Andersen’s model to further explain how perceived attractiveness of institutional care affects service utilization [14]. With the behavioral model, researchers can comprehensively review the related factors in utilization of long-term care services [15,16,17]. These studies found that none of these three factors can be left out of an analysis of what determines LTC service utilization, especially the need factor.

While there is an increasing emphasis on person-centered health care or LTC, phase 4 of Anderson’s health behavioral model could also provide a correspondent hypothesis. It suggests that researchers take the impact of care systems and customer satisfaction into account when they investigate the dynamic relationship between service utilization and health improvement [18]. According to Anderson’s model, a satisfactory experience can be attributed to continuity of services [8,18]. Positive perceptions of care could enhance access to quality care and in turn lead to better continuity of care among those already accessing to resources [19].

### 1.3. The Significance of the Long-Term Care Dataset and Previous Analysis

Since health status and functional needs are important predictors of LTC utilization and the changes in long-term trends also need to be considered in an evaluation of the system [20], a nationwide dataset or generational data (longitudinal analysis) is necessary when observing and monitoring care needs. This allows for determination of a causal relevance of health status and medical care utilization to be more representative [18]. There are studies analyzing the effectiveness of LTC systems using nationally-established datasets [21,22,23]. These results could provide an empirical basis for specific adjustments to policies and resource allocation. 

When LTC policy 1.0 was launched in 2008, Taiwan had already established a dataset, the LTC-Care Management Information System (LTC-CM). The dataset has been maintained by care managers in each county based on nationwide standardized information records that include physical, psychological, and social aspects of health indicators for LTC recipients. In the initial need assessments and reassessments, the care managers made subsequent care plans based on which home- and community-based service care recipients had actually received.

Few previous studies have analyzed information abstracted from the LTC-CM in Taiwan. A study confirmed a high case closure rate of 41% for those receiving home services in LTC plan 1.0 during the follow-up period in a southern city, for which the factors influencing case closure included non-low household incomes, high informal caregiving burden, and moderate levels of cognitive impairment [24]. Another study found caregivers of those with mild dementia may delay seeking medical advice for themselves. Therefore, policies should provide more respite services intended to meet the needs of caregivers [25].

### 1.4. Equity of Those Disadvantages in LTC Policy 1.0 

Health inequity is usually caused by social disadvantages related to the finances, gender, race, or ethnicity of those under consideration. To achieve health equity requires measures such as enhanced access to services or the elimination of unhealthy living or working conditions in these groups.

In terms of LTC, the low-income status of the elderly is typically associated with lower levels of life satisfaction, higher mortality rates, poor nutritional status, lower health literacy, and limited access to medical care when compared to higher-income individuals [26,27,28]. HCBS is designed to supplement the inadequacy of informal care and may assist with home-bound or low-income elderly aging in place [29]. Empirical research has shown that HCBS improves the mental and physical functioning and mental well-being of vulnerable groups [30,31].

However, the affordability of services to recipients or their informal caregivers remains a universal obstacle in accessing formal LTC services since household income is the strongest predictor of availability, accessibility, and affordability as they relate to service utilization in addition to need factors [32,33]. Hence, LTC should have a gradual payment system, require lower payment ratios for lower income families, and protect the poor from catastrophic medical and care costs [34].

Taiwan’s LTC policy does have gradual subsidy levels categorized by a means test. The direct effect of the policy is reduced willingness to use services in non-low-income household families [35]. As a consequence, whether LTC plan 1.0 achieves health equity by facilitating service accessibility is also an important issue.

### 1.5. Knowledge Gap, Study Aim, and Questions

Considering the relative lack of domestic research examining LTC plan 1.0 with quantitative data accumulated by the LTC-CM dataset nationwide, it is unclear whether the policy reform is based on a correct understanding of current problems. Therefore, this study aims to explore the effectiveness of HCBS utilization and its related influencing factors. It will also examine the equity issue of utilization by different social welfare groups under LTC policy 1.0. This work will add scientific support for policy making during the ongoing LTC reform.

## 2. Materials and Methods 

### 2.1. Data Sources and Samples

In this study, we analyzed the LTC dataset for 19 counties in Taiwan by using the Andersen and Aday’s behavioral model of healthcare utilization. Three counties on offshore islands were excluded due to the great difference in LTC resources. Data was tracked from 2013 to 2015 and was censored on 31 December 2016 if the case did not come to closure during the study period. After data cleaning, a total of 101,457 care recipients remained after selection from the LTC-CM dataset.

### 2.2. Outcome Variable

In the study, the utilization period was chosen as the outcome variable, that is, the length of time each care recipient received HCBS in the LTC system, from the initial need assessment to case closure or the censor date (31 December 2016) as measured by month. This variable represents the time of stay in LTC and the level of satisfaction with service utilization.

### 2.3. Independent Variables

The variables obtained from the dataset were classified into predisposing, enabling, need, and contextual factors, according to Anderson’s health behavioral model as follows:

1. Predisposing factors: age, gender, and education.
2. Enabling factors: living status, social welfare status, and primary caregiver.
3. Need factors: comorbidity, body mass index (BMI), dependency level using the Barthel Index [36], the IADL (measured using the Instrumental Activities of Daily Living) [37], depression (measured using the Center for Epidemiologic Studies Depression Scale (CES-D)) [38], and mental status (measured using the Short Portable Mental State Questionnaire (SPMSQ)) [39].
4. Contextual factors: level of service resources using a government survey to categorize as sufficient / insufficient resource area [40], proportion of certified nursing aides (CNAs), and district (categorized using all 19 counties into Northern/Central/Southern and Eastern, following the definition from the Council for Economic Planning and Development, Executive Yuan).

### 2.4. Statistical Analyses

**Figure 1:**
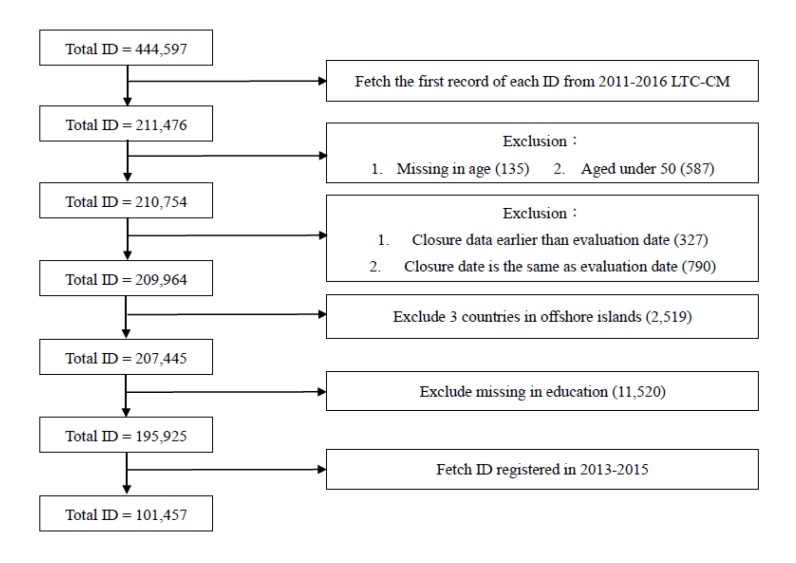
Flow chart of the exclusion process. **ID:** Identification number for citizens in Taiwan

The demographic variables are presented in the descriptive statistics. Referring to previous literature and the results of the univariate analysis, we identified the predictors of LTC service utilization with predisposing, enabling, need, and contextual factors. We conducted multiple imputations (MIs) to handle the missing data. All exclusion processes are shown in Figure 1. Finally, a multiple regression analysis was used to explore the factors influencing utilization of HCBS under LTC policy 1.0. Analyses were conducted with SPSS version 20.0 (SPSS Inc., Chicago, IL, USA) and SAS software, version 9.4 for Windows (SAS Institute Inc., Cary, NC, USA). 

## 3. Results

### 3.1. HCBS Utilization and Factors Influencing Usage

All demographic variables and related information are shown in Table 1.

A total of 101,457 eligible care recipients were in the national LTC-CM dataset from 2013 to 2015. The number of care recipients had grown steadily from 30,820 in 2013 to 35,049 in 2015. However, the utilization period became shorter during that period. In the study, only about 40.7% of the care recipients stayed in the care system for more than two years.

The gender proportion in the study was balanced, with women accounting for 54.15%. The age distribution was mainly concentrated in those aged 70–79 (32.41%) and those aged 80–89 (38.63%). Regarding education, nearly 36% of the care recipients were illiterate and recipients having more than seven years of education comprised less than one-third (26.36%) of the study sample. Only 13.72% of the care recipients lived alone. Generally, non-low-income households accounted for 82.18% in terms of their social welfare status and 12.24% of the care recipients didn’t have a primary caregiver. Within the caregiving relationship, adult children (41.52%) typically took on the responsibility of caring for their parents.

Most of the care recipients in the dataset had a body mass index of less than 24 (69.17%). In terms of the difficulties in performing activities of daily living (ADL), mild disability accounted for most cases (41.64%), followed by severe disability (33.13%), and moderate disability (20.51%), with only 4.73% of care recipients independent in terms of ADL. As for IADL, it was found that those with high function accounted for 48.38%, and those with low function accounted for 51.62%. About 4.5% of the care recipients had depressive tendencies and 45.05% of the care recipients had no mental impairments. Nearly two thirds (64.29%) of the care recipients reported three or more chronic conditions.

In the dataset, about one in ten (10.69%) of the care recipients lived in insufficiently resourced areas or aboriginal areas. About one third (35.62%) of the care recipients lived in the Northern District, 29.39% lived in the Central District, and 34.97% lived in the Southern and Eastern districts (Table 1).

The results of the multiple regression analyses are shown in Table 2. The significant predictors of a shorter HCBS utilization period included being male (β = −1.52), older (β = −0.08), lower IADL function (β = −0.7), having depression (β = −0.92), living in general service resources area (β = −0.86), and living in an area with a high proportion of CNAs (β = −0.24).

Recipients who tended to stay longer in the LTC system included those living in lower income households (β = 1.81, 1.83), those who had a primary caregiver (β = 1.16, 0.54, 0.85, 0.7), those with higher BMIs (β = 1.35), people with lower dependency levels (β = 1.75, 2.76, 3.49), and individuals with better mental status (β = 0.53, 0.61, 0.94).

The impact of education on the utilization period was inconsistent. It was more likely for recipients with 1–6 years of education to leave the LTC system than those who were illiterate (β = −0.2), with this tendency reversing among those having ≥7 years of education (β = 0.37).

### 3.2. Equity of HCBS Utilization under Different Social Welfare Statuses

To explore the social welfare status differences in terms of predicting utilization of HCBS, the regression model was further stratified by social welfare status, as shown in Table 3.

The results showed that gender, age, having a primary caregiver, BMI, dependency levels, IADL function, and mental status were all significant influencing factors in each household group, while depression, service resources level, and proportion of CNAs affected those in non-low-income households. Those with more prevalent signs of depression, more sufficient resources, a higher proportion of CNAs in their area, and a non-low-income household remained in the LTC system for shorter periods of time.

## 4. Discussion

### 4.1. The User Profile for Those Receiving HCBS

Based on the information for the 101,457 eligible recipients in the LTC-CM from 2013 to 2015, it was found that nearly half of the recipients were mildly dependent. Compared with the findings of a previous study analyzing users receiving home services in Taiwan, where the distribution of mild, moderate, and severe levels of disability were 33%, 24%, and 43%, respectively [24], the national profile seemed to reflect even lower levels of disability. In terms of social welfare status, this proportion was significantly different from the national survey in 2013, which stated that middle–low income accounted for 1.2%, and low-income accounted for 1.7% among total households. Therefore, people living in the government-subsidized HCBS system under the LTC 1.0 policy tend to be more economically disadvantaged. This situation was also found in terms of education level, as 35.98% of people living in the HCBS system were illiterate. However, according to the 2013 National Senior Citizen Condition Survey, elderly people aged 55 and over who were illiterate accounted for only 12.58%, those with 1–6 years of education accounted for 35.25%, and those with ≥7 years of education accounted for 52.17%.

In general, we found that the physical and mental functions of HCBS recipients in this study were better than expected. For example, recipients with mild ADL dependency (46.37%) or those who ranged from being cognitively intact to mild impairment (59.04%) were in the majority. However, under the LTC policy 1.0 era, they might have had lower socio-economic status and illiteracy rates might have been higher than the national census statistics indicated. The results did reflect the goal of the LTC 1.0 policy to take care of those who are disadvantaged. However, it also showed that most care recipients are those with less dependency levels in general.

### 4.2. HCBS Utilization Period and Influencing Factors

In terms of utilization, more than half of the care recipients left the services within two years. This result is similar to previous research that focused on home services in a city in southern Taiwan [41]. In our study, it was found that gender, age, education, social welfare status, having a primary caregiver, BMI, dependency level, depression symptoms, IADL functions, mental status, service resources level, and proportion of CNAs significantly affected the HCBS utilization time period based on the regression analysis. These results were mostly consistent with the findings of previous studies [16,17,42,43].

Being female is a factor that increases the possibility of use of long-term services. This phenomenon may be related to women being more likely to seek out health care services regardless of their health status [44,45,46].

The relation of education with the utilization period had opposite directions in different categories. Previous studies also observed inconsistent results related to education levels [12,24,47,48]. One possible explanation is that education is completed in young adulthood, but people typically start receiving care services once they are more mature. Hence, the relationship with service utilization or utilization period will tend to be inconsistent [42]. In addition, as a consequence of policy attention to vulnerable groups, the distribution of education levels in the study sample is relatively low compared with the Report of the National Senior Citizen Condition Survey 2013. Thus, the inconsistency observed in this study may have been related to the recruitment preferences for HCBS in the first place and may have further affected the perceptions of recipients and their families.

ADL dependency level may affect one’s living arrangements and then further affect choices related to use of long-term care services [49,50]. Disabled seniors need much more support and protection from their family or society than their younger counterparts, so older individuals with higher dependency levels are more likely to live with a spouse or adult children or to seek HCBS [51]. The positive impact of co-residence with informal caregivers on HCBS utilization also confirms that these types of services could supplement care needs in home settings [48]. According to previous studies, people with poor health status (referring to need factors) will begin to use informal care or HCBS. However, this study showed that they are also the ones leaving the LTC system faster than others even if they have had accessibility to formal care provided by the government. This phenomenon seems to not be what policymakers expected.

Due to the limited quality of the variable “cause of case closure” in the LTC-CM, it is difficult to clarify the exact reasons why recipients left the system. However, an earlier study shed light on one of the possible reasons which is referral to institutional care [52]. Generally speaking, the LTC system often uses hospitalization, mortality, and institutional replacement as quality analysis indicators, and these indicators are also reasons for discontinuation of HCBS use [53]. In the case of Taiwan, there might be an option of hiring 24 hour foreign caregivers to take care of senior relatives in their own home. Since the government liberalized foreign caregivers as a resolution for insufficient service resources and care manpower in 1992, the number of foreign caregivers has grown rapidly to 220,000 (National Statistics, 2020), indicating that foreign caregivers have become the most common choice for families in need. However, a domestic study found that cases accepting care from family members had less risk of hospitalization than those accepting care from foreign caregivers [54]. Therefore, the tendency of a shorter HCBS utilization period among recipients with severe physical and mental disabilities seemed to indicate that HCBS in LTC plan 1.0 failed to extend the time disabled older individuals could stay in their own communities and accept a high quality of care.

Moreover, a negative effect of environmental factors is not something policy makers want to see. The results indicate that the spatial distributions of service providers or quality of care manpower in HCBS in LTC plan 1.0 didn’t meet the expectations of recipients and their families. In fact, the goal of establishing adequate fundamental facilities such as adult daycare centers was not achieved until 2016 [5], and the numerous community care centers developed at the early stage of LTC plan 2.0 were allocated in an unbalanced manner [55].

Despite the fact that the LTC-CM did not collect alternative service information, it is conceivable that the spatial distributions of these service providers or caretakers would mostly be the same as formal care services provided by the government. Therefore, seeking alternative services for recipients living in areas with sufficient resources or higher proportions of CNAs would be easier than in areas without these amenities. Since LTC plan 2.0 is in the process of modification, a more accurate assessment method that takes unit distances, village populations, suppliers, and walkability for the elderly into account has been suggested as part of inventory resources [55]. This would ensure that areas that are currently insufficient will be covered in the future.

As for quality improvements in front-line manpower, areas that are the operative educational institution for LTC workers have regulated professional qualifications and on-the-job training specifications [56]. However, conventional training on unidisciplinary knowledge and skills is not enough to cope with the increasing complicated health and care needs inherent in LTC. Since LTC plan 2.0 aims at fostering person-centered care, team care courses would focus on decreasing fragmented communication among multi-disciplinary teams related to care delivery, and changes should be made in the culture of professional education in order to improve safety and quality of care [57].

Setting up strategies for improving the quality of manpower is consistent with suggestions made in previous studies, which found the productivity of the LTC system in Taiwan is mainly driven by technological changes [58]. Therefore, developing innovative measures to elevate quality of care is an urgent task for policy reform to improve the productivity of the LTC system.

### 4.3. Equity in the HCBS Utilization Period

Similar to education, the proportion of low-income households in this study sample was higher than in the national census statistics. Table 2 shows that individuals in mid–low income and low-income households tend to stay longer in the LTC system.

For those low-income recipients with low education levels, having an informal caregiver is not always helpful since their family may also have limited knowledge and lack of applicable care capabilities. Also, both economic and care burdens could exhaust these caregivers. Under these circumstances, they have no choice but to use HCBS as the most appropriate support [26,59]. However, the results of the regression model stratified by social welfare status demonstrated that need factors are always the strongest predictors of HCBS utilization duration regardless of income level, where recipients with higher dependency levels will leave the LTC system anyway. This indicates that in spite of care managers’ preferences to issue more subsidized hours to those from mid–low and low income households for severely dependent individuals [24], the intensity of formal care is still not adequate to meet their needs.

The impact of environmental factors found in this study was not unexpected and many countries have encountered this issue [32,33,60]. The different impact of environmental variables on the HCBS utilization period in the two social welfare status groups may have been derived from the ability to choose alternative care resources such as institutional care or foreign caregivers, as mentioned above, where without subsidies from the government, these services might be unaffordable to low income household families. Even institutional financial coverage could reduce financial barriers to LTC services, but organizational and geographic barriers will persist unless policy makers explicitly address these problems [61]. In order to ensure that recipients’ needs are met after enrolling in the LTC system and that services are accessible rather than simply visible, a more accurate resource inventory assessment method must be developed and policies must be developed and stipulated that encourage all types of service resources to settle in areas currently not covered [55].

In general, the analysis of factors influencing the HCBS utilization period among different social welfare status groups showed that there is not much disparity. However, it seems that the LTC plan 1.0 has yet to exert an actual protective effect on vulnerable groups, particularly in terms of their needs.

### 4.4. Limitations

This study based on the LTC-CM data set has the following limitations. Firstly, the analytical variables are confined within the dataset. In the current study, neither customer satisfaction nor health outcomes in vulnerable groups who left the system were traced. Some variables with missing data were not analyzed in our regressions since there was no information regarding imputation, although multiple imputations (MIs) have been conducted to handle the missing data. This means our results should be interpreted with caution. Determining whether health inequities can be eliminated after the system has reached out to recipients is also a crucial issue in social welfare programs and the sustainability of the LTC system. Furthermore, adding caregiver information into the analytical model in future studies would be helpful to gain a more comprehensive understanding of the outcome and performance of the LTC policies.

## 5. Conclusions

Based on an empirical analysis of a nationally representative dataset accumulated from the front line, this study was an attempt to provide evidenced-based knowledge and modification suggestions for LTC plan 2.0.

For long-term care needs, the phenomenon of short utilization period of the formal HCBS provided by the government was of concern. The two regression models revealed that the common factors influencing the length of the HCBS utilization period included need factors, where more dependent recipients will leave the LTC system regardless of their socio-economic status. However, the utilization period of non-low-income household recipients is significantly affected by the level of service resources, while those living in mid–low and low-income households are not affected.

To achieve the goal of the original policy which is aging in place, the system must reconsider care capacity and quality to keep moderate to severe care recipients with LTC needs from leaving the system. The LTC plan 2.0 reform service items have been increased, which includes more professional services, higher service density, and additional caregiver support services. However, it is still important to measure and monitor these changes in order to measure feedback and to reinforce the system. This in turn will help care recipients continue to live in their home communities which will achieve the goal of the aging in place policy.

## Figures and Tables

**Table 1:** Descriptive statistics of study samples who entered the HCBS formal system from 2013 to 2015.

Independent Variables	Categories	2013		2014		2015		Total	
		*n* = 30,820		*n* = 35,588		*n* = 35,049		*n* = 101,457	
		*n*	%	*n*	%	*n*	%	*n*	%
Utilization period	<6 months	4790	15.54%	5993	16.84%	5975	17.05%	16,758	16.52%
	≥24 months	13,601	44.13%	14,700	41.31%	384	1.10%	28,685	28.27%
Gender	Female	16,702	54.19%	19,264	54.13%	18,974	54.14%	54,940	54.15%
	Male	14,118	45.81%	16,324	45.87%	16,075	45.86%	46,517	45.85%
Age	50–59	1996	6.48%	2238	6.29%	2109	6.02%	6343	6.25%
Education	Illiteracy	11,426	37.07%	12,646	35.53%	12,429	35.46%	36,501	35.98%
	1–6 years	11,391	36.96%	13,544	38.06%	13,281	37.89%	38,216	37.67%
	≥7 years	8003	25.97%	9398	26.41%	9339	26.65%	26,740	26.36%
Living status	Alone	4315	14.00%	4833	13.58%	4772	13.62%	13,920	13.72%
	Co-residence	26,505	86.00%	30,755	86.42%	30,277	86.38%	87,537	86.28%
Social welfare status	Non-low	25,258	81.95%	29,216	82.10%	28,905	82.47%	83,379	82.18%
	Mid-low	2224	7.22%	2612	7.34%	2472	7.05%	7308	7.20%
	Low income households	3330	10.80%	3755	10.55%	3669	10.47%	10,754	10.60%
Primary caregiver	No	3760	12.20%	4363	12.26%	4293	12.25%	12,416	12.24%
	Spouse	8644	28.05%	9684	27.21%	9381	26.77%	27,709	27.31%
	Daughter-in-law	3395	11.02%	3847	10.81%	3705	10.57%	10,947	10.79%
	Children	12,500	40.56%	14,856	41.74%	14,770	42.14%	42,126	41.52%
	Others	2521	8.18%	2838	7.97%	2900	8.27%	8259	8.14%
BMI	BMI < 24	21,333	69.22%	24,603	69.13%	24,240	69.16%	70,176	69.17%
	BMI ≥ 24	9487	30.78%	10,985	30.87%	10,809	30.84%	31,281	30.83%
Dependency levels	Severe	10,308	33.45%	11,837	33.26%	11,464	32.71%	33,609	33.13%
	Moderate	6213	20.16%	7377	20.73%	7215	20.59%	20,805	20.51%
	Mild	12,920	41.92%	14,658	41.19%	14,665	41.84%	42,243	41.64%
	Independent	1379	4.47%	1716	4.82%	1705	4.86%	4800	4.73%
IADL	High function	14,732	47.80%	17,070	47.97%	17,284	49.31%	49,086	48.38%
	Low function	16,088	52.20%	18,518	52.03%	17,765	50.69%	52,371	51.62%
Depression	No	29,254	94.92%	34,006	95.55%	33,634	95.96%	96,894	95.50%
	Yes	1566	5.08%	1582	4.45%	1415	4.04%	4563	4.50%
Mental status	Intact	13,934	45.21%	16,235	45.62%	15,541	44.34%	45,710	45.05%
	Mild imp.	4328	14.04%	4960	13.94%	4904	13.99%	14,192	13.99%
	Moderate imp.	5177	16.80%	5842	16.42%	6112	17.44%	17,131	16.88%
	Severe imp.	7243	23.50%	8417	23.65%	8301	23.68%	23,961	23.62%
Comorbidity	0	622	2.02%	527	1.48%	642	1.83%	1791	1.77%
	1	3546	11.51%	3895	10.94%	3690	10.53%	11,131	10.97%
	2	7225	23.44%	8233	23.13%	7846	22.39%	23,304	22.97%
	≥3	19,427	63.03%	22,933	64.44%	22,871	65.25%	65,231	64.29%
LTC service resources level	Insufficient area and aboriginal area	3314	10.75%	3762	10.57%	3770	10.76%	10,846	10.69%
	Sufficient area	27,498	89.22%	31,821	89.41%	31,274	89.23%	90,593	89.29%
District	Northern	10,596	34.38%	13,368	37.56%	12,170	34.72%	36,134	35.62%
	Central	9249	30.01%	10,311	28.97%	10,262	29.28%	29,822	29.39%
	Southern and Eastern	10,967	35.58%	11,904	33.45%	12,612	35.98%	35,483	34.97%

NOTE: *n* = 101,457 in the LTC-CM from 2013–2015. The numbers of new entry in each year were shown in the system. ADL disability was categorized into independent (scores > 90), mild disability (61 ≤ scores < 90), moderate disability (31 ≤ scores < 60), and severe disability (scores ≤ 30) according to the need assessment scale of LTC plan 1.0. IADL (Instrumental Activities of Daily Living) was categorized into high function was ≥8 points and low function was <8 points. Depression: CES-D ≥ 12 in male and ≥10 in female. No depression: CES-D < 12 in male and <10 in female). Mental status was categorized into intact/mild, impairment/moderate, and impairment/severe. Cognitive impairments measured by Short Portable Mental State Questionnaire (SPMSQ) scores were adjusted for education level. Care managers were evaluated in cases where individuals could not answer the SPMSQ themselves.

**Table 2:** The influencing factors of the HCBS utilization period as determined by multiple regression analyses.

Independent Variables	Categories		Model 1 (*n* = 101,457)		Model 2 (*n* = 101,441)		Model 3 (*n* = 100,985)		Model 4 (*n* = 100,983)	
Predisposing Factor										
Gender (Female)		Male	−1.62	***	−1.84	***	−1.60	***	−1.52	***
Age			−0.12	***	−0.09	***	−0.07	***	−0.08	***
Education (Illiteracy)		1–6 years	0.13	0.15	0.20	*	0.06	0.487	−0.20	*
		≥7 years	0.77	***	0.94	***	0.85	***	0.37	***
Enabling Factor										
Living status (Co-residence)		Alone			1.37	***	0.11	0.366	0.22	0.070
Social welfare status (Non-low)		Mid-low			1.63	***	1.74	***	1.81	***
		Low income households			1.76	***	1.87	***	1.83	***
Primary caregiver (No)		Spouse			0.76	***	0.74	***	1.16	***
		Daughter-in-law			−0.04	0.792	0.09	0.559	0.54	***
		Children			0.45	***	0.50	***	0.85	***
		Others			0.41	*	0.43	**	0.70	***
Need Factor										
BMI (<24)		BMI ≥ 24					1.34	***	1.35	***
Dependency levels (Severe)		Moderate					1.68	***	1.75	***
		Mild					2.69	***	2.76	***
		Independent					3.35	***	3.49	***
IADL (High function)		Low function					−0.07	0.370	−0.70	***
Depression (No)		Yes					−0.97	***	−0.92	***
Mental status (Severe imp.)		Mild impairment					0.49	***	0.53	***
		Moderate impairment					0.60	***	0.61	***
		Intact					1.03	***	0.94	***
Sufficient Resources Area									−0.86	***
CNAs ^#^ Proportion									−0.24	***
Adjusted R-squared			0.116		0.12		0.137		0.144	

Notes: * *p* < 0.05; ** *p* < 0.01; *** *p* < 0.001. ^#^ CNAs stands for Certified Nursing Assistants working in HCBS. Comorbidity, district (Northern, Central, Southern and Eastern), and the year care recipients registered in the LTC-CM (2013, 2014, 2015) were used as control variables in this model. Utilization period was measured by month.

**Table 3:** Influencing factors of HCBS utilization stratified by social welfare status.

Independent Variables	Non-Low-Income Households			Mid-low Income and Low-Income Households	
	*n* = 82,926			*n* = 18,057	
Gender (Female)	Male	−1.54	***	−1.40	***
Age		−0.07	***	−0.10	***
Education (Illiteracy)	1–6 years	−0.08	0.403	−0.90	*
	≥7 years	0.49	***	−0.62	0.448
Living status (Co-residence)	Alone	0.28	*	0.02	0.393
Primary caregiver (No)	Spouse	0.91	***	2.11	***
	Daughter-in-law	0.29	0.106	1.22	***
	Children	0.62	***	1.26	***
	Others	0.34	0.097	1.33	***
BMI (<4)	BMI ≥ 24	1.36	***	1.29	***
Dependency level (Severe)	Moderate	1.62	***	2.55	***
	Mild	2.53	***	3.96	***
	Independent	3.25	***	4.58	***
IADL (High function)	Low function	−0.65	***	−1.03	***
Depression (No)	Yes	−0.91	***	−0.98	0.194
Mental status (Severe impairment)	Mild impairment	0.53	***	0.76	*
	Moderate impairment	0.52	***	1.25	*
	Intact	0.84	***	1.69	***
Sufficient resources area		−0.91	***	−0.51	0.208
CNAs proportion		−0.22	***	−0.30	0.312
Adjusted R. squared		0.128		0.202	

Notes: * *p* < 0.05; *** *p* < 0.001. Comorbidity, district (Northern, Central, Southern and Eastern) and the year care recipients registered in LTC-CM (2013, 2014, 2015) were used as control variables in this model. Utilization period was measured by month.
